# A Manual of Surgery for Students and Practitioners

**Published:** 1899-07

**Authors:** 


A Manual of Surgery for Students and Practitioners.—By William Rose, M. B., B. S., London, F. R. C. S., Professor of Clinical Surgery, Kings College, London, and Senior Surgeon to Kings College Hospital, etc., and Albert Carless, M. S., London, F. R. C. S., Senior Assistant Surgeon in Kings College, London, etc. Published by William Wood & Co., New York. 1898. Price, cloth, $5.00, net.

This is a compact and handy volume of 1160 pages, and fully justifies its authors' claim, that it meets a genuine need, viz: to meet the wants of the medical student and general practitioner. By omitting all theoretical, experimental and bibliographical matter, as well as all subjects which are now almost universally conceded to the various specialties, the size of the volume is reduced to reasonable bounds, and yet space is left sufficient for an ample discussion of the subjects of particular interest to the practitioner and student.

The work is not a reprint of a stale foreign work interlarded with some few dozen notes by an American editor, but is fresh from the English as well as the American press, having been published in England in May, 1898. It may be taken as a valuable and accurate reflection of the surgical science of our English cousins, and is certainly both timely and creditable. J.

